# The Critical Role of Tetrahydrobiopterin (BH4) Metabolism in Modulating Radiosensitivity: BH4/NOS Axis as an Angel or a Devil

**DOI:** 10.3389/fonc.2021.720632

**Published:** 2021-08-27

**Authors:** Yang Feng, Yahui Feng, Liming Gu, Pengfei Liu, Jianping Cao, Shuyu Zhang

**Affiliations:** ^1^School of Radiation Medicine and Protection, State Key Laboratory of Radiation Medicine, Soochow University, Suzhou, China; ^2^China National Nuclear Corporation 416 Hospital (Second Affiliated Hospital of Chengdu Medical College), Chengdu, China; ^3^West China Second University Hospital, Sichuan University, Chengdu, China; ^4^West China School of Basic Medical Sciences and Forensic Medicine, Sichuan University, Chengdu, China

**Keywords:** radiation, nitric oxide synthase (NOS), nitric oxide (NO), 5, 6, 7, 8-tetrahydrobiopterin (BH4), radiosensitivity

## Abstract

Ionizing radiation and radioactive materials have been widely used in industry, medicine, science and military. The efficacy of radiotherapy and adverse effects of normal tissues are closed related to cellular radiosensitivity. Molecular mechanisms underlying radiosensitivity are of significance to tumor cell radiosensitization as well as normal tissue radioprotection. 5,6,7,8-Tetrahydrobiopterin (BH4) is an essential cofactor for nitric oxide synthases (NOS) and aromatic amino acid hydroxylases, and its biosynthesis involves *de novo* biosynthesis and a pterin salvage pathway. In this review we overview the role of BH4 metabolism in modulating radiosensitivity. BH4 homeostasis determines the role of NOS, affecting the production of nitric oxide (NO) and oxygen free radicals. Under conditions of oxidative stress, such as UV-radiation and ionizing radiation, BH4 availability is diminished due to its oxidation, which subsequently leads to NOS uncoupling and generation of highly oxidative free radicals. On the other hand, BH4/NOS axis facilitates vascular normalization, a process by which antiangiogenic therapy corrects structural and functional flaws of tumor blood vessels, which enhances radiotherapy efficacy. Therefore, BH4/NOS axis may serve as an angel or a devil in regulating cellular radiosensitivity. Finally, we will address future perspectives, not only from the standpoint of perceived advances in treatment, but also from the potential mechanisms. These advances have demonstrated that it is possible to modulate cellular radiosensitivity through BH4 metabolism.

## Radiation-Induced Injuries and ROS Generation

Ionizing radiation and radioactive materials have been widely used in industry, medicine, science and military. In addition, widespread application of nuclear technology may increase accidental or occupational radiation exposure, such as nuclear accidents, terrorist attacks, etc, which finally leads to radiation-induced injury or even mortality (1, 2). Radiosensitivity determines the injury severity or even survival exposed to ionizing radiation. Radiotherapy is an indispensable component of malignancy treatment, either alone or in combination with other treatments (3), which is applied to over 50% of all cancer patients (4). Although the accuracy of radiotherapy is improving, normal tissues are more or less damaged, resulting in toxicity, which may be a critical dose-limiting complication and affect the quality of life (5–7). Numerous approaches to modulate radiosensitivity, including increasing tumor response to radiotherapy and minimizing damage to normal tissues, have been reported in the past decade (8). Unfortunately, there has been a dearth of clinical treatments for radiation-induced injuries. Various compounds have been identified as potential radiation protection agents, such as free radical scavengers, antioxidants, cytokines, etc (9, 10). Amifostine is a FDA-approved radioprotector, an effective free radical scavenging agent (11), and has been extensively studied and used in clinical radiotherapy (12). It is worth noting that amifostine cannot protect all human organs from the toxic effects of ionizing radiation (13) and it has obvious side-effects, such as nausea and vomiting, which may cause its discontinuation during radiotherapy (14, 15). Thus, it is essential to uncover the mechanisms underlying cellular radiosensitivity and to innovate alternative agents with radioprotective or/and radiosensitization properties in clinical applications.

Ionizing radiation induces cellular damage through direct deposition of energy and indirect oxidative damage caused by excessive reactive oxygen species (ROS), which is the main toxic effects of ionizing radiation (16). Radiation-induced accumulation of ROS results in protein, lipid and DNA damage, leading to a series of pathophysiological changes and ultimately to acute and/or chronic damage (17). Microvascular injury is a distinctive feature of acute and chronic radiation injury. The dysfunction of vascular endothelium caused by ionizing radiation play a crucial role in the occurrence and development of radiation damage (18, 19). Radiation exposure can induce different degrees of functional and morphological changes in vascular endothelial cells, including apoptosis, loss of thrombus resistance and increased endothelial permeability. Endothelial nitric oxide synthase (eNOS) plays an important role in radiation injury. Radiation exposure impairs the function of eNOS and inhibits the production of endothelia nitric oxide (NO) (20). Collectively, strategies to prevent or ameliorate post-radiation endothelial dysfunction may improve the severity of radiation injury.

## Functional Significance of BH4

**Figure 1:**
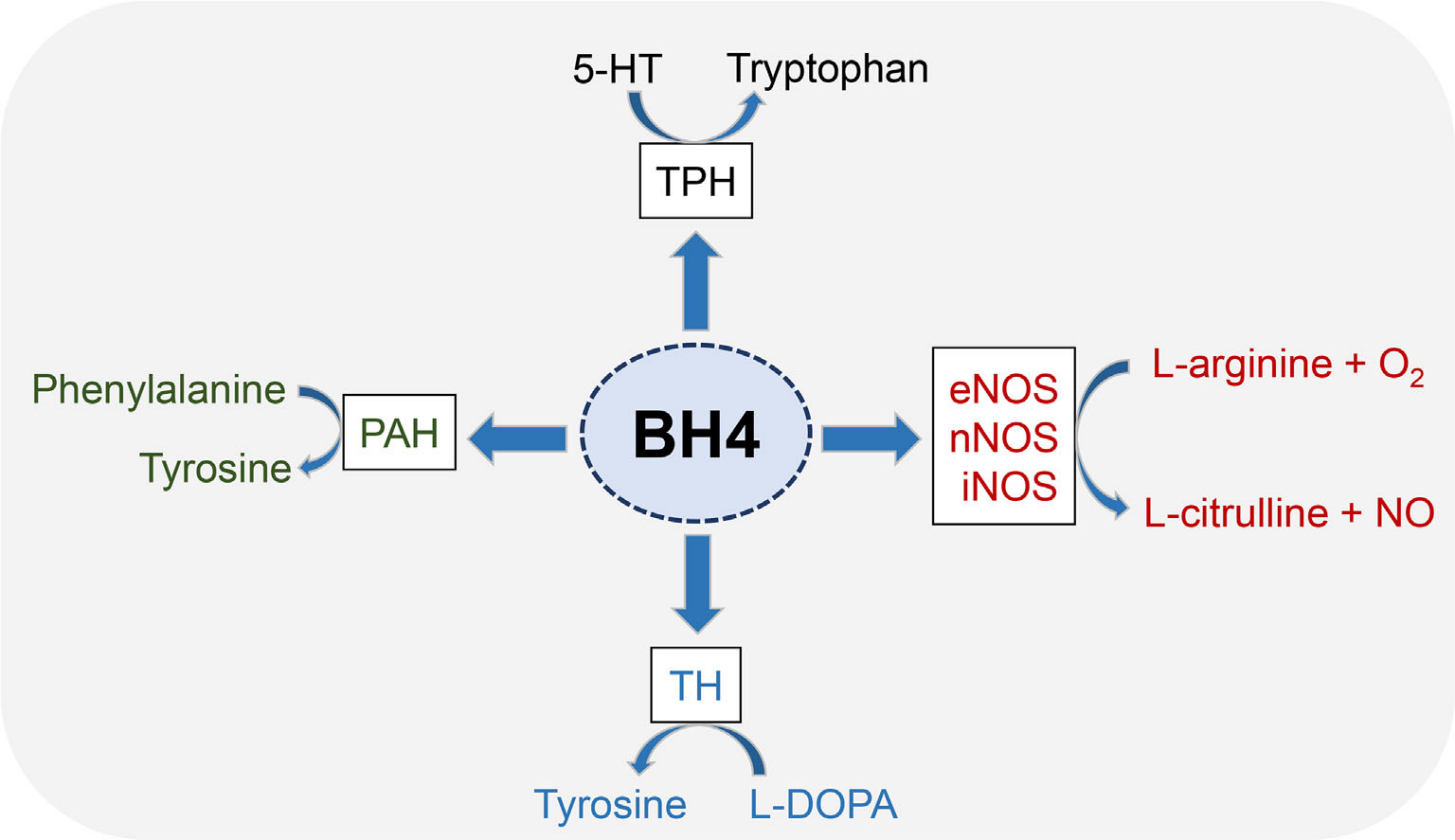
Enzyme cofactor activity of BH4. BH4 is an essential cofactor for multiple enzymes, including three aromatic amino acid hydroxylases (PAH, TH and TPH) and nitric oxide synthases (NOSs). All three NOSs need BH4 to produce NO. Generally, these enzymes combine the oxidation of L-arginine with the reduction of molecular oxygen to form NO and L-citrulline. BH4 is also the cofactor activity of three aromatic amino acid hydroxylases, which leads to the synthesis of neurotransmitters and prevents the accumulation of phenylalanine.

5,6,7,8-tetrahydrobiopterin (BH4) is an essential cofactor for multiple enzymes, including three aromatic amino acid hydroxylases (phenylalanine hydroxylase, tyrosine hydroxylase and tryptophan hydroxylase) and nitric oxide synthases (NOSs) (**Figure 1**). Phenylalanine hydroxylase (PAH) is first enzyme recognized as a BH4-dependent enzyme (21). The activity of rat liver PAH is disrupted by ionizing radiation, thereby exerting a negative effect on BH4 activity (22, 23). Other aromatic amino acid hydroxylases, such as tyrosine hydroxylase (TH) and tryptophan hydroxylase (TPH), share common features with PAH with respect to the reaction mechanism (24), to BH4 (25) and substrate binding (26).

NOSs includes endothelial nitric oxide synthase (eNOS), neuronal nitric oxide synthase (nNOS) and induced nitric oxide synthase (iNOS) (27), wherein eNOS has been shown to play a key role in radiation damage and has been emerging as a therapeutic target (28). NOS catalyzes the conversion of L-arginine to L-citrulline and NO (29). NOS dimers consist of two identical monomers and each monomer consisting of a C-terminal reductase domain and an N-terminal oxygenase domain. The C-terminal binding flavin mononucleotide, flavin adenine dinucleotide and NADPH. The N-terminal binding sites containing heme, BH4, and L-arginine (20). BH4 has been proven to regulate NOS functions at a variety of levels. BH4 enhances NOS enzyme activity by increasing heme iron levels (30) and increases the affinity of NOS with its substrate (31). In addition, BH4 can promote the stability of NOS dimer structure, which is essential for NOS function (32).

## Biosynthesis and Regulation of BH4

**Figure 2:**
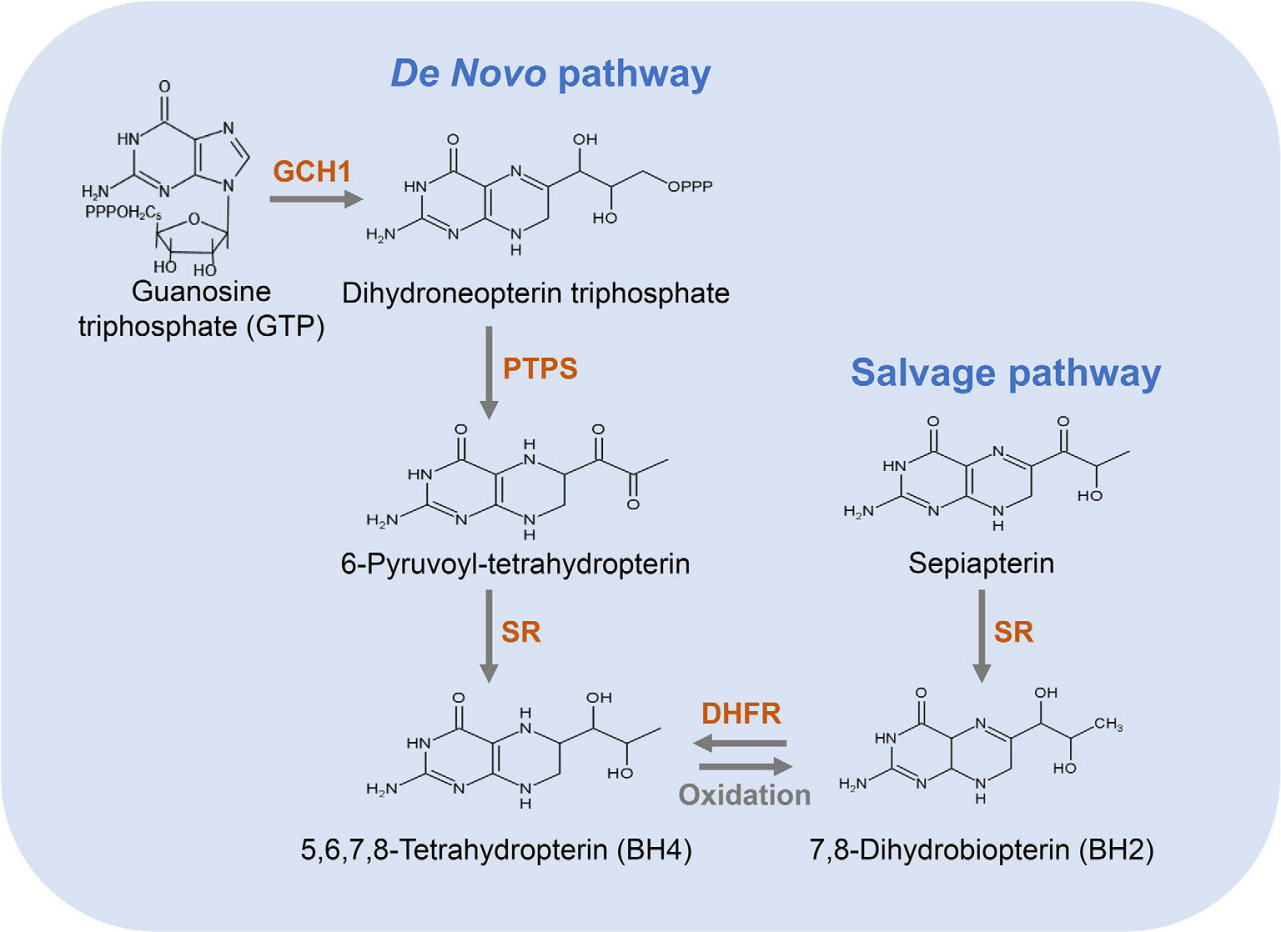
Pathways for the biosynthesis of BH4. The *de novo* pathway (left) is synthesized from GTP to BH4 in three steps. GCH1 is the rate-limiting enzyme in BH4 *de novo* biosynthesis. The salvage pathway (right) produces BH4 from its oxidized form, starting with sepiapterin in two steps, which is essential to convert exogenous sepiapterin into BH4.

There are two distinct pathways for BH4 biosynthesis, including *de novo* pathway and salvage pathway (33) (**Figure 2**). The former refers to the synthesis of BH4 from guanosine triphosphate (GTP) through three enzymatic reactions; the latter refers to the process of converting sepiapterin as a substrate to BH2 and further reduction to BH4 (20). The relative contribution of the *de novo* and salvage pathways to the cellular availability of BH4 varies depending on the cell type.

### *De Novo* Pathway of BH4 Synthesis

In the *de novo* pathway, BH4 is synthesized from GTP by three enzymes, namely GTP cyclohydrolase I (GCH1), 6-pyruvyl-tetrahydrobiopterin synthase (PTPS) and methotrexate reductase (SR). As shown in **Figure 2**, GCH1 is the rate-limiting enzyme in BH4 *de novo* biosynthesis, which catalyzes the formation of dihydroneopterin triphosphate (DNTP). The first step in the synthesis of BH4 is complicated and highly regulated at the level of transcription, translation and post-translation (34). Next, DNTP is converted into 6-pyruvyl-tetrahydrobiopterin by PTPS. Although the key rate-limiting enzyme in the *de novo* synthesis pathway of BH4 is GCH1 in most cells, PTPS has also been considered as a rate-limiting enzyme in certain types of cells, especially in human liver cells (35). After being stimulated by cytokines, LPS, hydrogen peroxide, insulin and other immune stimuli, GCH1 expression is induced and PTPS therefore becomes a rate-limiting enzyme (35). In the final step of this pathway, SR catalyzes the production of 6-pyruvyl-tetrahydrobiopterin to BH4. This step involves two consecutive NADPH-dependent reduction reactions. The side chain carboxyl of 6-pyruvyl-tetrahydrobiopterin is first reduced and rearranged to form the intermediate 6-lactanoyl-tetrahydrobiopterin, which is then reduced to BH4 on the second side chain carboxyl (35).

As a key enzyme for *de novo* pathway of BH4 synthesis, GCH1 activity is regulated by various factors, such as transcriptional factors, post-translational regulation, and activity regulatory proteins. In particular, we have demonstrated that AU-rich element RNA-binding factor 1 (AUF1) regulates GCH1 expression *via* its 3’UTR (36). Phosphorylation is a common post-translational modification in organisms (37). Post-translational regulation of GCH1 activity appears to occur by the protein phosphorylation. It has been reported that phosphorylation of GCH1 at serine 81 is critical in the activation of this enzyme because it not only improves its intrinsic activity and increases its protein expression level, but also reduces the feedback inhibition of its regulatory protein GTP cyclohydrolase I feedback regulator (GFRP) (38–40). The interaction between GCH1 and GFRP can either activate or inhibit GCH1 activity (41). In the presence of phenylalanine, GFRP interacts with GCH1 to activate the GCH1 activity (42, 43). In contrast, the binding of GCH1 with GFRP mediates the feedback inhibition of BH4 (42).

### Salvage Pathway of BH4 Synthesis

The salvage pathway generates BH4 from its oxidized forms under the action of sepiapterin reductase (SR) and dihydrofolate reductase (DHFR) (**Figure 2**). SR is a homodimer composed of two subunits, which takes part not only in the *de novo* synthetic pathway of BH4 but also in the salvage biosynthetic pathway (44). Additionally, many non-pteridine derivatives, vicinal dicarbonyls, monoaldehydes and monoketones are sensitive as substrates of SR (44). It has been found that the regulation of endothelial BH4 content is mainly accomplished through salvage pathway (45, 46) and the decrease in SR leads to an impairment in endothelial BH4. Similarly, an increase in SR leads to an increase in BH4 level and NO production, and a reduction in oxygen radical production. DHFR is an enzyme necessary for the biosynthesis of folate in eukaryotic and prokaryotic cells (47). In addition to crucial roles in folate metabolism, DHFR can also reduce BH2 and thus regenerate BH4 (48). Previous studies have shown that DHFR plays a key role in determining BH4 homeostasis, NO bioavailability and NOS coupling in endothelial cells (49). When endothelial cells are stimulated *via* angiotensin II, DHFR expression is down-regulated, BH4 level is decreased, and NOS uncoupling is increased, which is restored by DHFR overexpression (45). Thus, DHFR is crucial in maintaining endothelial BH4 levels and NO bioavailability under oxidative stress.

The salvage pathway is very essential for the conversion of sepiapterin to BH4. Although sepiapterin is not a metabolite of mammals, it is a key exogenous substance that enhances BH4 levels in mammals (50). Thus, supplementation of cells with sepiapterin has been a common strategy to increase intracellular BH4 levels *via* the salvage pathway.

## The Effect of Radiation on BH4 Metabolism and Possible Molecular Signaling Pathways

BH4 is reductive and easily oxidized to BH2 when damaged, such as UV-radiation and ionizing radiation. Oxidation of BH4 to BH2 and other oxidized biopterin species causes eNOS to produce higher superoxide levels instead of NO, a phenomenon commonly referred to eNOS, leading to increased oxidative stress (20, 51). Ionizing radiation oxidizes BH4. Engin et al. found that the urinary biopterin concentration is significantly higher in radiation-exposed hospital staff compared with the healthy subjects (52). BH4 plasma level is significantly lower in patients with abdominal radiotherapy one week after radiotherapy (53). Similarly, after daily exposure to 4 Gy, the plasma BH4 level of the rats decreases significantly, which is consistent with the downward trend of the plasma BH4 level of the patients receiving abdominal radiotherapy. Compared with wild-type mice, BH4 deficient mice show an increase in radiation-induced aortic peroxynitrite in lung tissues (54). Radiation-induced salivary gland dysfunction in mice is attributed to increased peroxynitrite (55). All these data indicate that ionizing radiation promotes the formation of peroxynitrite, which is likely to be the result of reduced BH4 availability after radiation (56).

GFRP overexpression increases the interaction between GFRP and GCH1, thereby negatively regulating the biosynthesis of BH4 and increasing the level of oxidative stress induced by ionizing radiation (54). The mRNA expressions of GFRP in lung and liver of wild- type mice increase after 8.5 Gy of total body Irradiation (TBI), suggesting that the inhibition of GCH1 activity mediated by GFRP may be a possible mechanism of BH4 inhibition after ionizing irradiation (54, 57).

iNOS activity is activated immediately after ionizing radiation (within 2 h) *via* NF-κB pathway (58), thereby inducing NO production, which may then interact with radiation-induced superoxide to form peroxynitrite (56). Peroxynitrite is prone to oxidize BH4, implying that the NF-κB pathway plays a key role in modulating the bioavailability of BH4 after ionizing radiation (59). Fascinatingly, coordinated activation of JAK-STAT pathway and NF-κB pathway may be involved in radiation-induced BH4 deficiency (56, 60).

Despite the finding that BH4 metabolism involves in NOS uncoupling and ROS production, the effect of radiation on BH4 metabolism may have other mechanisms. It has been reported that protein S-nitrosylation, an important post-translational modification (61, 62), requires NO participation. Dysregulated S-nitrosylation has been shown in multiple human diseases (63, 64). Microbiome-derived NO promotes extensive S-nitrosylation of the host proteome to regulate miRNAs, gene expression as well as host functions and physiology (65). BH4 production mediated by PTPS facilitates latent TGF-β binding protein 1 (LTBP1) S-nitrosylation, thereby suppressing TGF-β secretion and promoting tumor growth (66). Components of the ubiquitin-proteasome system are altered by BH4-dependent NO signaling *via* protein S-nitrosylation, which implicates the widespread impact of BH4 on downstream cellular signaling (67). Recent studies have delineated a previously unrecognized link between BH4 metabolism and ferroptosis (68), which is associated with radiotherapy (69). So dysregulated S-nitrosylation may also be responsible for the reduction of BH4 after irradiation.

## BH4 and eNOS Function

NO is a potent endogenous vasodilator produced by NOS. eNOS produces NO which is an essential regulator of endothelial function, participating in various physiological events and is a key regulator of endothelial cell migration, survival and angiogenesis (70). The impaired NO production by eNOS is a main reason for endothelial dysfunction (71, 72). Reduced NO production in diabetic patients is associated with the pathogenesis of endothelial dysfunction (73, 74). Multiple studies have demonstrated that ionizing radiation inhibits the activity of eNOS and reduces the production of endothelial NO. Even years after radiotherapy, there is still endothelial dysfunction in the increased expression of pro-thrombotic and pro-inflammatory markers in irradiated blood vessels (75). Functional eNOS oxidizes L-arginine to L-citrulline and NO in the presence of BH4, which is an effective natural reducing agent (76). Suboptimal levels of BH4 due to the oxidation to BH2 *via* stimuli such as radiation exposure may lead to NOS uncoupling and the subsequent generation of highly oxidative radicals, including superoxide and peroxynitrite (77), which is the main mechanism of impaired vascular regulation (78). When BH4 is limited, activated NOS cannot catalyze the conversion of L-arginine to L-citrulline and NO, but can still accept electrons from NADPH and transfer electrons to another substrate O_2_, resulting in the production of O_2_^-^ instead of NO. BH4 is oxidized by ONOO^-^ to BH2 and then to biopterin (B). BH2 together with NOS causes ROS production instead of NO (20, 51). Thus, similar to BH4, BH2 has an affinity for the pterin-binding site, which makes it an efficient uncoupling agent for NOS (79). NOS activity is strictly regulated by plenty of biochemical pathways (80), including the availability of its cofactor BH4 (81). For example, compared with age-matched females, the higher oxidative stress of male spontaneously hypertensive rats leads to the relative lack of BH4, leading to the decrease of renal NOS activity and NO bioavailability (82–85).

## BH4 Metabolism and Ionizing Radiation

### BH4 Metabolism and Radiation-Induced Injuries

Since BH4 reduces ROS by regulating NOS product (86–88), BH4 has been shown to play a key role in the pathogenesis of multiple diseases characterized by increased oxidative stress, such as diabetes, arteriosclerosis, hypertension and radiation-induced injuries (48, 53, 89, 90). Stress-induced ROS production may reduce the availability of BH4, which may induce NOS uncoupling and increase the production of oxidative superoxide radicals. NOS can catalyze the production of NO and L-valine from L-arginine in the presence of sufficient BH4 (19, 20, 48). BH4 is likely to be involved in free radical production and may be related to the progression of radiogenic damage. So far, multiple studies have focused on the biochemical and mechanistic effects of BH4 in radiation-induced injuries and the radioprotective effect of BH4 has been confirmed (53, 90). It has been shown that radioprotection of BH4 through some mechanisms such as scavenging free radicals, promoting responses to DNA damage, and alleviating inflammatory responses, etc (53, 90).

BH4 has become a potential strategy for fibrosis and diastolic dysfunction, which are all related to ROS (91, 92). We have previously reported that GCH1 expression and BH4 levels in irradiated human skin and rat skin tissues are lower than that in the unirradiated counterparts, which impairs NO homeostasis and enhances ROS cascade (90). Oxidative stress-responsive transcriptional factor Nrf2 is able to transcriptionally activate GCH1, thereby restoring cellular BH4 level and attenuating procession of radiation-induced skin injury *in vitro* and *in vivo* (90).

BH4 treatment can decrease oxidative stress in irradiated cardiomyocytes, thereby reducing radiation damage and improving myocardial function (93). NO is insufficient after ionizing radiation, which is one of the key indicators of myocardial fibrosis. Patients with fibrotic diseases show low NO levels (94, 95). It has been reported that BH4 supplementation can restore NO and reduce animal myocardial fibrosis (96). BH4 can inhibit the decoupling of NOS and improve cardiac dysfunction (59, 79, 97–100). One month after the aortic arch narrowing of C57 mice, NOS decoupling and oxidative stress occur, exogenous administration of BH4 can improve myocardial function. When the coronary artery is severely narrowed, perfusion of BH4 can improve its diastolic function (101). GCH1 activity and BH4 level are decreased in irradiated mesenteric artery and endothelial cells. Administration of a GCH1 inhibitor DAHP significantly aggravates vascular injury and intestinal damage, while BH4 treatment can improve intestinal vascular injury and ischemia induced by ionizing radiation, and restore vascular function (53). Recent study has shown that the co-administration of Sildenafil (SD) and simvastatin (SV), NO donor/BH4 regulator, inhibits the cranial irradiation-induced oxidative stress, inflammation, NO-pathway dysregulation and neuronal apoptosis, indicating a neuroprotective effect role of SD/SV in irradiation-induced brain injury as a possible mechanism of its NO donor/BH4 regulatory activities (102).

GT3, a radioprotective vitamin E analog, can reduce radiation-induced oxidative/nitrosative stress (89). GT3 regulates the expression of GFRP and thus plays its radioprotective role in part by regulating the BH4 availability (89). A *GFRP*-overexpressing transgenic mice display reduced tissue BH4 and blood GSH levels, indicating a higher oxidative stress (54). Cheema et al. investigated liver metabolic changes following irradiation in control and *GFRP* overexpression mice (57). Compared with wild-type mice, *GFRP* transgenic mice show reduced glutathione levels and increased levels of glycocholic acid and N-arachidonic taurine after irradiation, suggesting the early occurrence of metabolic dysfunction. Thus, *GFRP* transgenic mice are susceptible to radiation stress and this sensitivity may lead to increased radiation-induced injuries (54).

**Figure 3:**
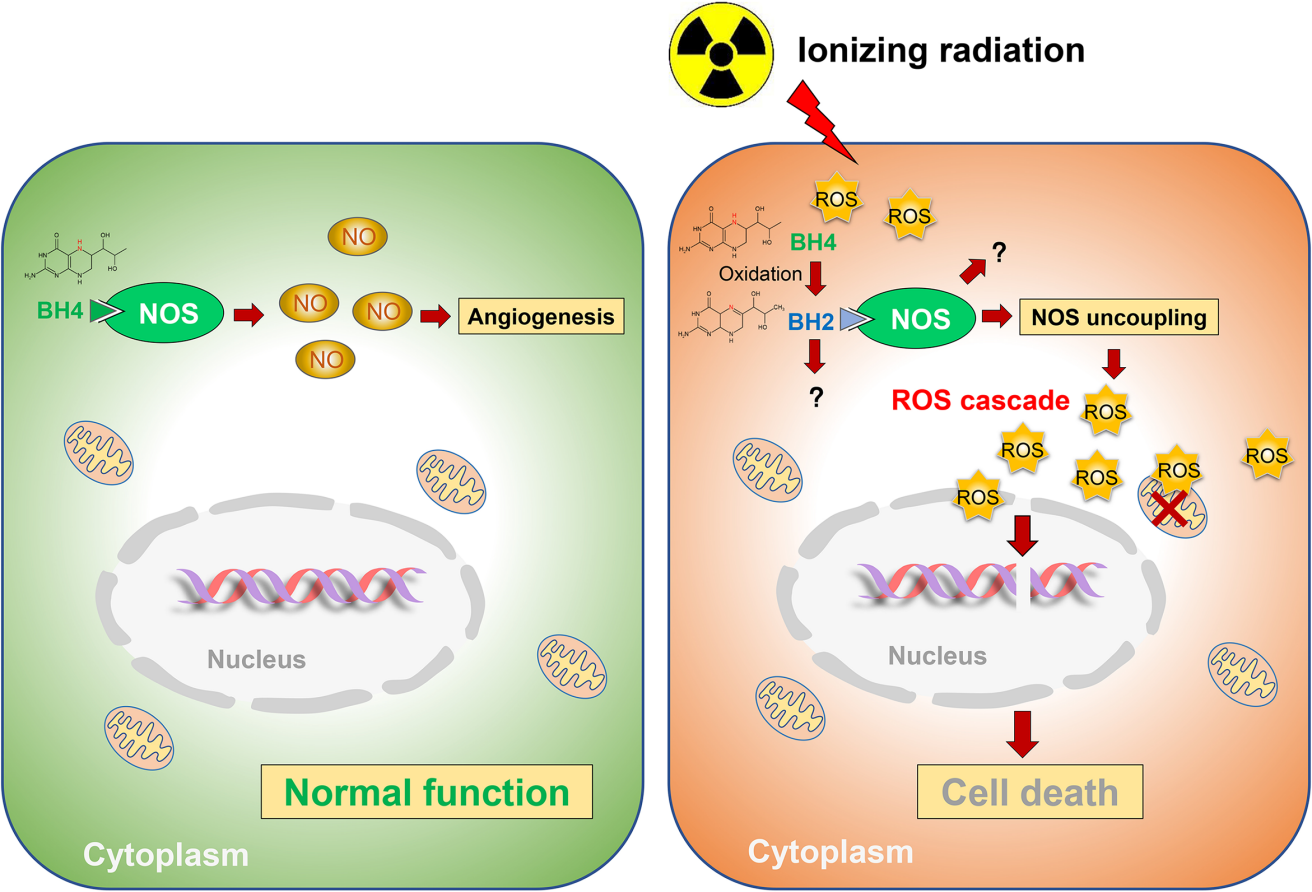
Schematic representation of BH4 metabolism in radiosensitivity. Radiation oxidates BH4, which results in NOS uncoupling and augmented radiation-induced secondary ROS, ultimately leading to radiation-induced injuries. While GCH1-mediated BH4 metabolism attenuated radiation-induced ROS production to improve radiation damage.

Collectively, ionizing radiation oxidizes BH4, which results in NOS uncoupling and augmented radiation-induced secondary ROS, ultimately leading to radiation-induced injuries. While GCH1-mediated BH4 metabolism attenuates radiation-induced ROS production to improve radiation damage (**Figure 3**).

### BH4 Metabolism and Cancer Radiosensitivity

**Figure 4:**
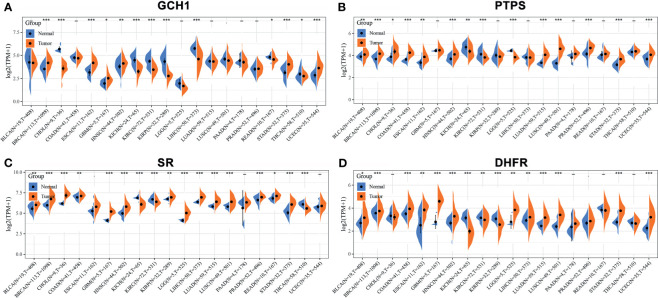
The expression of BH4 metabolic enzymes in human tumors. The comparison of **(A)** GCH1, **(B)** PTPS, **(C)** SR and **(D)** DHFR expression in in various tumor tissues and corresponding normal tissues. And BH4 metabolic enzymes generally overexpress in tumor tissues than corresponding normal tissues. *P < 0.05, **P < 0.01 and ***P < 0.001, compared with the normal tissues. Gene expression data are obtained from the Cancer Genome Atlas (TCGA) database (44). BLCA, Bladder urothelial carcinoma; BRCA, Breast invasive carcinoma; CHOL, Cholangiocarcinoma; COAD, Colon adenocarcinoma; ESCA, Esophageal carcinoma; GBM, Glioblastoma multiforme; HNSC, Head and Neck squamous cell carcinoma; KICH, Kidney Chromophobe; KIRC, Kidney renal clear cell carcinoma; KIRP, Kidney renal papillary cell carcinoma; LGG, Brain Lower Grade Glioma; LIHC, Liver hepatocellular carcinoma; LUAD, Lung adenocarcinoma; LUSC, Lung squamous cell carcinoma; PAAD, Pancreatic adenocarcinoma; PRAD, Prostate adenocarcinoma; READ, Rectum adenocarcinoma; STAD, Stomach adenocarcinoma; THCA, Thyroid carcinoma; UCEC, Uterine Corpus Endometrial Carcinoma.

We retrieved the expression of BH4 metabolic enzymes (GCH1, PTPS, SR and DHFR) in various tumors based on the Cancer Genome Atlas (TCGA) database (**Figure 4**) (44). According to the results, GCH1 exhibits relatively higher expression levels in the liver, endometrium and breast cancers than the tumor adjacent tissues. PTPS is overexpressed in lung, colon and endometrium cancers. The expression of the SPR gene is higher in liver cancer, colorectal cancer and invasive breast carcinoma. And DHFR gene is highly expressed in glioblastoma multiforme, invasive breast carcinoma, stomach adenocarcinoma and uterine corpus endometrial carcinoma. These different BH4 metabolic enzymes may be related to specific tissue functions. In a word, BH4 metabolic enzymes are generally overexpressed in tumor tissues than in corresponding normal tissues, which may be due to the higher ROS level in tumor cells (103). Therefore, BH4 metabolic enzymes are possible hallmarks and therapeutic targets.

In addition to the radioprotective effects of BH4 and its metabolites, some studies have shown that they can improve the therapeutic effect of radiotherapy. Therefore, BH4 and its metabolites are considered as radiosensitization targets in cancer radiotherapy. It has been reported that BH4/BH2 ratio in colorectal, breast and head and neck tumors is significantly lower than that in normal tissues (104). In mouse spontaneous breast cancer model, exogenous BH4 precursor sepiapterin increases BH4/BH2 ratio, which enhances the NOS activity and increases NO production. Sepiapterin finally leads to the transition from pro-inflammatory/pro-survival signals to anti-inflammatory/pro-apoptotic signals, thereby inhibiting spontaneous tumor growth (104). In addition, in a murine SCCVII tumor model, radiation-induced NO through increases eNOS activity mitigates tumor hypoxia and increases radiosensitivity (105). It is now clear that NO, which is associated with malignancy, may exhibit a dual activity: stimulating tumor growth and having the opposite anti-tumor effect (106), which depends on the concentration of NO (107, 108). At low concentrations, NO can inhibit apoptosis and cause mutations, which may lead to the formation of malignant growth loci. Conversely, high concentrations of NO seem to be harmful to malignant cells, especially when exposed to ionizing radiation (107–109). Kashiwagi et al. (110) demonstrated that NOS activity affects tumor blood vessels. Inhibition of NOS in glioma cells can improve oxygen delivery and a more normal phenotype (110). Whereas, vasculature normalization with antiangiogenics is short-lived. Treatment of mice with the NOS inhibitor L-NNA reduces tumor blood flow, resulting in delayed tumor growth, but quickly lost its effect (111). On the other hand, post ionizing radiation NOS inhibition delays tumor growth *via* Th1 immune polarization within the tumor microenvironment (112). A 6-day sepiapterin treatment in mice reduces tumor blood flow, delays tumor growth and improves animal survival, while tumor oxygenation continues to improve significantly after 10 days of sepiapterin treatment and improved tumor oxygenation is associated with increased tumor cell apoptosis (113). Pretreatment with sepiapterin not only enhances the killing of tumor by ionizing radiation, but also enhances the absorption of doxorubicin. Thus, as a vascular normalizing agent, sepiapterin can reduce tumor hypoxia, improve tumor %HbO2 and perfusion, and prevent cancer cells from acquiring aggressive phenotypes in the hypoxic microenvironment, ultimately leading to radiation-induced apoptosis, thereby enhancing tumor radio- and chemosensitivities (113, 114).

**Figure 5:**
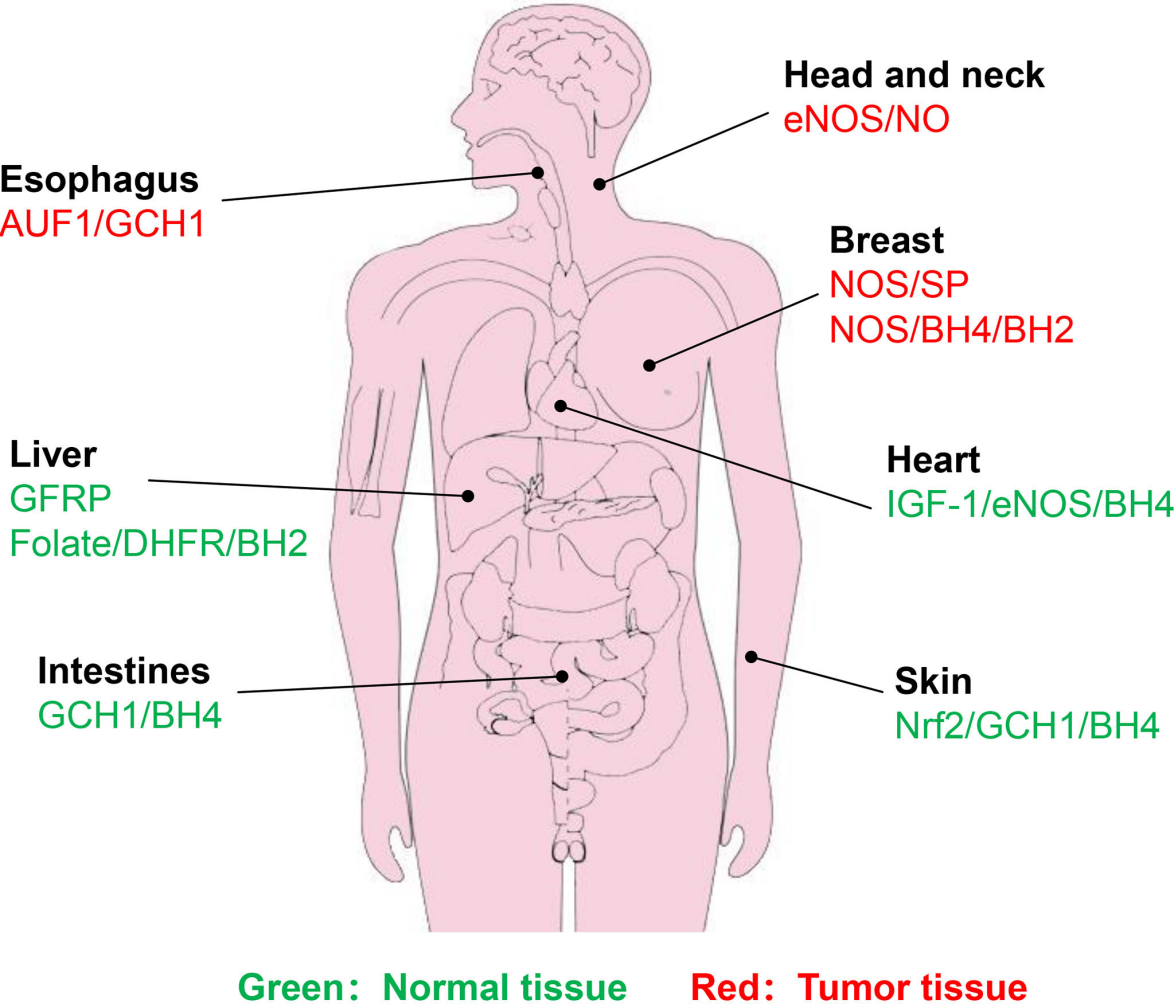
Identified normal tissues or cancer types associated with BH4-mdiated radiosensitivity. Green represents normal tissue, while red represents tumor tissue. Brief description of pathways associated with BH4 are shown.

In the salvage pathway, DHFR exhibits a critical role in BH4 generation. Radiotherapy, however, tends to trigger DHFR amplification, thereby enhancing the activity of DHFR (115, 116). Enhanced DHFR activity promotes DNA replication in cervical cancer cells, leading to reduced therapeutic efficacy (117). The use of DHFR inhibitors, such as methotrexate (MTX) analogues as radiosensitizers is expected to improve the therapeutic effect (118, 119). Liang et al. (117) synthesized a series of 2,4-diaminopteridine analogues as DHFR inhibitors for radiosensitization. In particular, the combination of X-rays and a compound named 2a effectively suppresses cervical tumor growth and compound 2a has higher radiosensitization activity than MTX. Hence, if the DHFR activity is inhibited, the radiotherapy effect will be improved to varying degrees. The normal tissue or cancer types associated with BH4-mediated radiosensitivity are summarized in **Figure 5**.

## Future Directions

Since BH4 is easily oxidated (20, 51, 120), novel approaches are needed to protect its integrity during delivery. Nanotechnology offers a new way to deliver drugs efficiently and specifically. It is reported that liposome formulations can improve the therapeutic effect of drugs with poor bioavailability (121). Liposomal BH4 has been used to reverse the loss of BH4 after ischemia-reperfusion injury (122, 123). Similarly, targeted delivery of BH4 nanocarriers can be used as a prophylactic treatment for atherosclerosis (124). Thus, it is possible to use novel approaches, such as liposomes and nanoparticles, to carry BH4 to enhance its stability and make its clinical application more promising. In addition, the design of ROS responsive nanomaterials based on the high ROS conditions at radiation-damaged sites provides a new approach for BH4 loading for radioprotection. Although it has been reported that vitamin C, folic acid, etc. can enhance the binding of BH4 to eNOS, thereby increasing the level of intracellular BH4 (48, 125), the clinical efficacy is compromised due to difficulties to combine with BH4. The rescue approach of regulating BH4 synthesis through its precursor sepiapterin may be another treatment strategy (126).

During cancer radiotherapy, the role of BH4 metabolism in cancer cell radiosensitivity yet to be determined. BH4 on one hand reduced radiogenic ROS, however, BH4 on the other hand normalizes vessels, which enhances radiotherapy efficacy. Given the rapid development of targeted therapies, specific radiosensitizers can be used for cancer radiotherapy. Tumor cells constantly interact with the surrounding microenvironment. Apart from the tumor cells, the tumor microenvironment includes a variety of cell types (endothelial cells, fibroblasts, immune cells, etc.) and extracellular components (cytokines, growth factors, hormones, extracellular matrix, etc.) (127). High expression of GCH1 in cancer-associated fibroblasts stimulates breast cancer cell proliferation and motility (128). As a critical T-cell regulator, BH4 can be manipulated to enhance immunity and inhibit tumor growth (129). The role of BH4 metabolism in tumor microenvironment is largely unknown. Further research on these mechanisms will accelerate the development of radiosensitizers based on BH4 metabolism.

## Author Contributions

YF and YHF drafted the manuscript and figures. LG and PL collected literature. JC and SZ modified the manuscript. All authors contributed to the article and approved the submitted version.

## Funding

This work is supported by the National Natural Science Foundation of China (U1967220, and 82073477), the Key Research & Development Program of Jiangsu Province (BE2020637), Post graduate Research & Practice Innovation Program of Jiangsu Province (No KYCX21_2969) and the Young Talent Project of China National Nuclear Corporation.

## Conflict of Interest

The authors declare that the research was conducted in the absence of any commercial or financial relationships that could be construed as a potential conflict of interest.

## Publisher’s Note

All claims expressed in this article are solely those of the authors and do not necessarily represent those of their affiliated organizations, or those of the publisher, the editors and the reviewers. Any product that may be evaluated in this article, or claim that may be made by its manufacturer, is not guaranteed or endorsed by the publisher.
